# Extending Three-Dimensional Weighted Cone Beam Filtered Backprojection (CB-FBP) Algorithm for Image Reconstruction in Volumetric CT at Low Helical Pitches

**DOI:** 10.1155/IJBI/2006/45942

**Published:** 2006-09-05

**Authors:** Xiangyang Tang, Jiang Hsieh, Roy A. Nilsen, Scott M. McOlash

**Affiliations:** GE Healthcare, 3000 North Grandview Boulevard, W-1190, Waukesha, WI 53188, USA

## Abstract

A three-dimensional (3D) weighted helical cone beam filtered backprojection (CB-FBP) algorithm (namely, original 3D weighted helical CB-FBP algorithm) has already been proposed to reconstruct images from the projection data acquired along a helical trajectory in angular ranges up to [0, 2 π]. However, an overscan is usually employed in the clinic to reconstruct tomographic images with superior noise characteristics at the most challenging anatomic structures, such as head and spine, extremity imaging, and CT angiography as well. To obtain the most achievable noise characteristics or dose efficiency in a helical overscan, we extended the 3D weighted helical CB-FBP algorithm to handle helical pitches that are smaller than 1: 1 (namely extended 3D weighted helical CB-FBP algorithm). By decomposing a helical over scan with an angular range of [0, 2π + Δβ] into a union of full scans corresponding to an angular range of [0, 2π], the extended 3D weighted function is a summation of all 3D weighting functions corresponding to each full scan. An experimental evaluation shows that the extended 3D weighted helical CB-FBP algorithm can improve noise characteristics or dose efficiency of the 3D weighted helical CB-FBP algorithm at a helical pitch smaller than 1: 1, while its reconstruction accuracy and computational efficiency are maintained. It is believed that, such an efficient CB reconstruction algorithm that can provide superior noise characteristics or dose efficiency at low helical pitches may find its extensive applications in CT medical imaging.


Hindawi Publishing Corporation
International Journal of Biomedical Imaging
Volume 2006, Article ID 45942, Pages 1–8
DOI 10.1155/IJBI/2006/45942

### Extending Three-Dimensional Weighted Cone Beam Filtered Backprojection (CB-FBP) Algorithm for Image Reconstruction in Volumetric CT at Low Helical Pitches

**Xiangyang Tang, Jiang Hsieh, Roy A. Nilsen, and Scott M. McOlash**

*GE Healthcare, 3000 North Grandview Boulevard, W-1190, Waukesha, WI 53188, USA*

Received 23 December 2005; Revised 24 May 2006; Accepted 25 May 2006

A three-dimensional (3D) weighted helical cone beam filtered backprojection (CB-FBP) algorithm (namely, original 3D weighted helical CB-FBP algorithm) has already been proposed to reconstruct images from the projection data acquired along a helical trajectory in angular ranges up to $[0, 2\pi]$. However, an overscan is usually employed in the clinic to reconstruct tomographic images with superior noise characteristics at the most challenging anatomic structures, such as head and spine, extremity imaging, and CT angiography as well. To obtain the most achievable noise characteristics or dose efficiency in a helical overscan, we extended the 3D weighted helical CB-FBP algorithm to handle helical pitches that are smaller than 1 : 1 (namely extended 3D weighted helical CB-FBP algorithm). By decomposing a helical over scan with an angular range of $[0, 2\pi + \Delta\beta]$ into a union of full scans corresponding to an angular range of $[0, 2\pi]$, the extended 3D weighted function is a summation of all 3D weighting functions corresponding to each full scan. An experimental evaluation shows that the extended 3D weighted helical CB-FBP algorithm can improve noise characteristics or dose efficiency of the 3D weighted helical CB-FBP algorithm at a helical pitch smaller than 1 : 1, while its reconstruction accuracy and computational efficiency are maintained. It is believed that, such an efficient CB reconstruction algorithm that can provide superior noise characteristics or dose efficiency at low helical pitches may find its extensive applications in CT medical imaging.

Copyright © 2006 Xiangyang Tang et al. This is an open access article distributed under the Creative Commons Attribution License, which permits unrestricted use, distribution, and reproduction in any medium, provided the original work is properly cited.

### 1. INTRODUCTION

Along with the fast evolution in theoretically exact helical cone beam (CB) reconstruction algorithms [1–5], in the recent years comes the exciting progress in theoretically approximate helical CB reconstruction algorithms [6–9]. A three-dimensional (3D) weighted helical CB filtered backprojection (CB-FBP) algorithm (namely, original 3D weighted helical CB-FBP algorithm) has been proposed to reconstruct images from the projection data acquired along a helical trajectory within an angular range up to $[0, 2\pi]$ [6]. Except for the adoption of 3D weighting functions, the original 3D weighted helical CB-FBP algorithm is essentially similar to the FDK algorithm [10] and its extensions [11]. By using phantoms simulated by computer and scanned by CB volumetric CT scanners, the reconstruction accuracy and other properties of the original 3D weighted helical CB-FBP algorithm have been experimentally evaluated and verified. Although it is theoretically approximate, the experimental evaluation shows that, at a moderate cone angle up to $4^\circ$ that corresponds to a detector $z$-dimension of $64 \times 0.625$ mm, the original 3D weighted helical CB-FBP algorithm reaches the reconstruction accuracy comparable to that of theoretically exact helical CB-FBP algorithms, such as the algorithm proposed by Katsevich [1, 2], and its extensions [3–5]. Moreover, other imaging performances, such as noise characteristics or dose efficiency, noise uniformity, spatial resolution, temporal resolution, computational efficiency, and robustness over clinical applications, are maintained comparable with the FDK-like CB reconstruction algorithms [10, 11].

A helical trajectory angular range of $[0, 2\pi]$ corresponds to a full scan [12, 13], under which the normalized helical pitch is usually about 1 : 1. However, a helical overscan is usually employed in the clinic to reconstruct tomographic images with superior noise characteristics at the most challenging anatomic structures, such as head and spine, extremity imaging, and CT angiography as well. In an overscan, the projection data acquired along a helical trajectory angular range larger than $[0, 2\pi]$ should be utilized to reconstruct an image, and the corresponding normalized helical pitch is usually smaller than 1 : 1. Due to the $[0, 2\pi]$ constraint in helical trajectory angular range, a direct application of the original 3D weighted helical CB-FBP algorithm in an overscan cannot make full use of available projection data, resulting in a degraded noise characteristics or dose efficiency.

To improve noise characteristics or dose efficiency in an overscan, we extended the original 3D weighted helical CB-FBP algorithm to handle helical pitches that are lower than 1 : 1 (namely extended 3D weighted helical CB-FBP algorithm). As in the original algorithm, the extended algorithm can be implemented in either the native CB geometry or the cone-parallel geometry (or wedge-geometry) that is obtained through row-wise fan-to-parallel rebinning from the native CB geometry. An experimental study is conducted in this paper to evaluate the reconstruction accuracy and noise characteristics or dose efficiency of the extended 3D weighted helical CB-FBP algorithm. Since image reconstructed in the cone-parallel geometry is of better noise uniformity and computation efficiency, the experimental evaluation is carried out in the cone-parallel geometry using the helical body phantom (HBP) [14, 15] and the Defrise phantom [16] simulated by computer. As shown below, at helical overscan, the extended 3D weighted helical CB-FBP algorithm can provide significantly improved noise characteristics or dose efficiency in comparison to the original 3D weighted helical CB-FBP algorithm, while other advantages of the original algorithm, such as reconstruction accuracy and computational efficiency, can be maintained.

### 2. MATERIALS AND METHODS

### 2.1. Native cone beam and cone-parallel geometries

The native cone beam geometry for the helical projection data acquisition and image reconstruction is shown in Figure 1(a), where $Oxyz$ denotes the coordinate system, $S$ the source focal spot, $D$ the cylindrical multirow CT detector, and $R$ the radius of the helical source trajectory. $P(x, y, z)$ is a point within the object to be reconstructed. The ray emanating from focal spot $S$ and passing through point $P(x, y, z)$ is uniquely determined by its view angle $\beta$, fan angle $\gamma$, and cone angle $\alpha$. The helical source trajectory can be mathematically represented by

$$\mathrm{ST}(\beta) = \left(R\sin\beta, R\cos\beta, \frac{H}{2\pi}\beta\right), \quad \beta \subseteq [\beta_s, \beta_e], \quad (1)$$

where $\beta_s$ and $\beta_e$ are the starting and ending points of the helical source trajectory, respectively. Notice that view angle $\beta$ is defined in relative to the $y$-axis, and $H$ is the distance traveled by the source focal spot per rotation along the $z$-axis.

Through row-wise fan-to-parallel rebinning in the native CB geometry, the cone-parallel geometry for image reconstruction is attained as shown in Figure 1(b) [17–19]. The ray emanating from focal spot $S$ and passing through point $P(x, y, z)$ is uniquely determined by its view angle $\beta$, orthogonal distance $t$ from the iso-ray (namely orthogonal iso-distance), and cone angle $\alpha$.

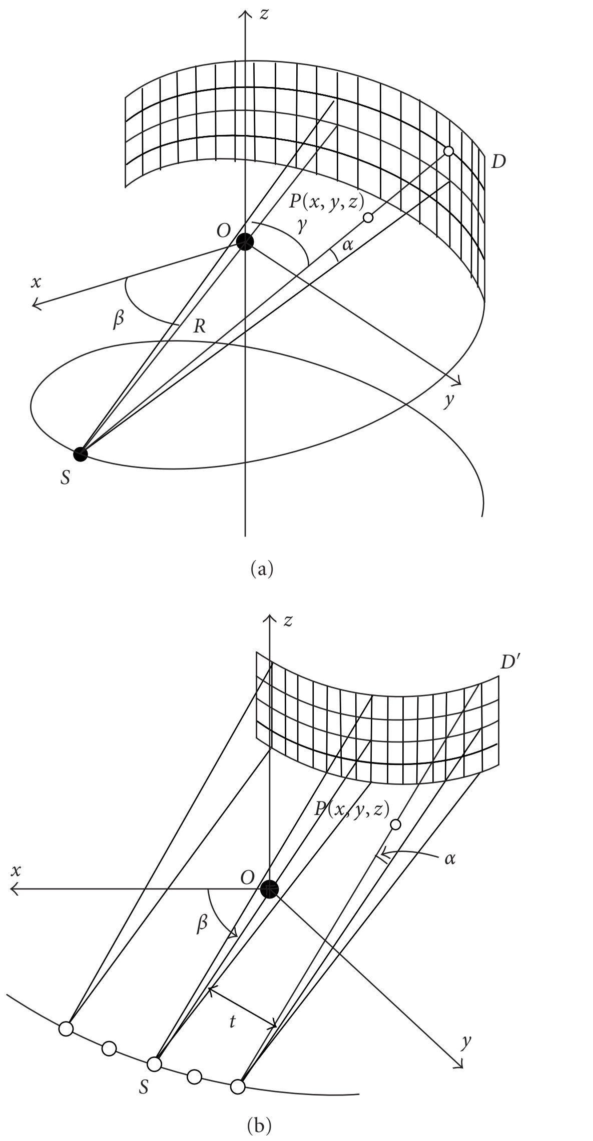

Figure 1: Schematic diagrams showing the geometries in which the extended 3D weighted helical CBFBP reconstruction algorithm is derived: (a) the native CB geometry; (b) the cone-parallel geometry.

### 2.2. 3D weighted CB-FBP reconstruction algorithm

In the cone-parallel geometry shown in Figure 1(b), the original 3D weighted helical CB-FBP reconstruction algorithm for a full scan is [6]

$$\tilde{f}(x, y, z) = \frac{1}{2}\int_{\beta_0-\pi}^{\beta_0+\pi} \frac{R}{\sqrt{R^2 + Z^2(x, y, x)}} w_{3d}(\alpha, \beta, t)\tilde{s}(\alpha, \beta, t)\, d\beta, \quad (2)$$

$$\tilde{s}(\alpha, \beta, t) = s(\alpha, \beta, t) \otimes q(t), \quad (3)$$

where $Z(x, y, z)$ is the projected $z$-coordinate of point $P(x, y, z)$ onto detector $D$, and $q(t)$ is the conventional ramp filter kernel in parallel beam geometry. The interval $[\beta_0 - \pi, \beta_0 + \pi]$ defines the view angle range over which the projection data are used to reconstruct an image intersecting the helical source trajectory at view angle $\beta_0$. It is important to note that the filtering in (3) is naturally tangential [6, 20] due to the row-wise fan-to-parallel rebinning.

The 3D weighting function can be expressed in the form

$$w_{3d}(\alpha,\beta,t) = \frac{w_{2d}(\beta,t)g(\alpha_c, p(h))}{w_{2d}(\beta,t)g(\alpha_c, p(h)) + w_{2d}(\beta_c,t_c)g(\alpha, p(h))}, \tag{4}$$

where $\alpha$ and $\alpha_c$ are the cone angles corresponding to a direct ray and its conjugate ray, respectively [6], and $h$ is the normalized helical pitch defined by

$$h = \frac{H}{L}, \tag{5}$$

where $L$ represents the height of the detector along $z$-direction at the iso-center.

$g(|\alpha|, p(h))$ is a monotonically increasing function over the magnitude of cone angle $\alpha$, that is, given a normalized helical pitch $h$, one has

$$g(|\alpha_1|, p(h)) < g(|\alpha_2|, p(h)), \quad \text{while } |\alpha_1| < |\alpha_2|, \tag{6}$$

where $p(h)$ is a monotonic increasing function of helical pitch $h$, that is,

$$p(h_1) < p(h_2), \quad \text{while } h_1 < h_2, \tag{7}$$

and the 3D weighting function $w_{3d}(\alpha,\beta,\gamma)$ has to satisfy the normalization condition

$$w_{3d}(\alpha,\beta,t) + w_{3d}(\alpha_c,\beta_c,t_c) = 1.0. \tag{8}$$

Any 3D weighting function $w_{3d}(\alpha,\beta,\gamma)$ satisfying the conditions specified by (6)–(8) is acceptable, and a special example is given by [6]

$$w_{3d}(\alpha,\beta,\gamma) = \frac{w_{2d}(\beta,t)\tan^{kh}(\alpha_c)}{w_{2d}(\beta,t)\tan^{kh}(\alpha_c) + w_{2d}(\beta_c,t_c)\tan^{kh}(\alpha)}, \tag{9}$$

where $k$ is a parameter that varies over different helical pitches, and can be adjusted to get a balanced capability between artifact suppression and noise characteristics. $w_{2d}(\beta,t)$ is a 2D view weighting function and can find its examples in the literature [12, 13, 21–24].

### 2.3. *Projection of reconstruction plane and 3D weighting*

Prior to extending the original 3D weighted helical CB-FBP algorithm presented above, it is insightful for us to investigate the projection of reconstruction plane in helical scanning in the cone-parallel geometry. Supposing the reconstruction plane is orthogonal to the $z$-axis and intersects the helical source trajectory at $\beta = 0°$, shown in Figure 2(a) are the projections of reconstruction plane at helical pitch 63/64 : 1 corresponding to view angle $\beta = -180°, -90°, -45°, 0°, 45°, 90°, 180°$, respectively, in which the white area corresponds to the projection of the reconstruction plane, and the grey area the outside of the detector. At such a helical pitch, about half projection of the reconstruction plane at $\beta = \pm180°$ is outside the detector boundary (detector $z$-dimension: $64 \times 0.625$ mm), and this is the reason why 3D weighting is needed in the original 3D weighted helical CB-FBP algorithm to reconstruct images in a full scan [6]. However, at a lower helical pitch, such as 33/64 : 1 as shown in Figure 2(b), projections of the reconstruction plane at $\beta = \pm180°$ are within the detector boundaries. Notice that, even at $\beta = \pm240°$, a larger portion of the projection of reconstruction plane at pitch 33/64 : 1 is within the detector boundaries than that at helical pitch 63/64 : 1. Hence, it is intuitive to deduce that projection data corresponding to an overscan can be utilized at helical pitch 33/64 to reconstruct images with better noise characteristics or dose efficiency if the original 3D weighted helical CB-FBP algorithm can be extended to deal with overscan.

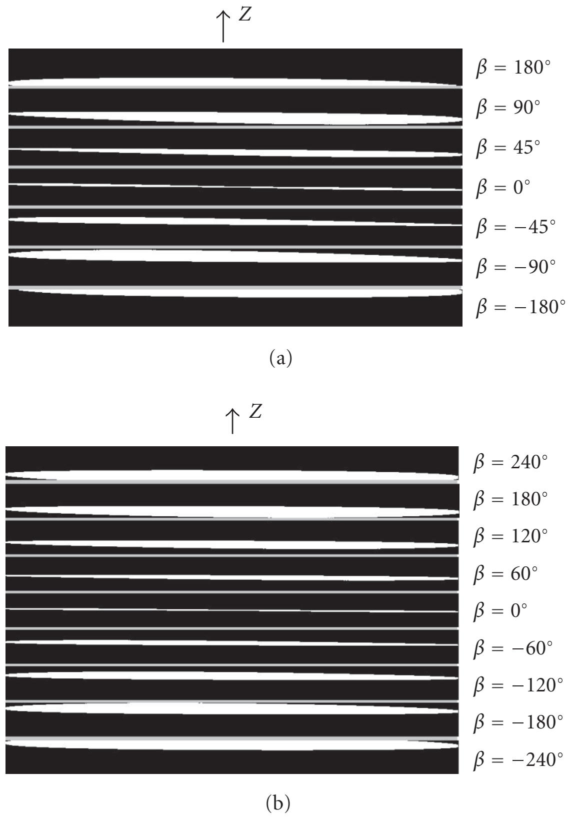

Figure 2: The schematic diagram showing the projection of image plane as a function over view angle at (a) helical pitch 33/64 : 1; and (b) helical pitch 63/64 : 1. (The vertical direction is parallel to the rotation axis of a CT gantry, while the horizontal direction corresponds to the latitudinal direction of the gantry.)

### 2.4. Extended 3D weighted helical CB-FBP algorithm

Suppose the angular range of projection data corresponding to an overscan is

$$[\beta_{\min}, \beta_{\text{axn}}] = [0, 2\pi + \Delta\beta]. \tag{10}$$

A direct extension of the original algorithm by just stretching the angular range from $[0, 2\pi]$ to $[0, 2\pi + \Delta\beta]$ violates the normalization condition specified in (8) and can result in artifacts in reconstructed images. However, if the helical trajectory angular range of an overscan $[0, 2\pi + \Delta\beta]$ is decomposed into a union of $N$ overlapped subangular ranges

$$[\beta_{\min}, \beta_{\max}] = \bigcup_{i=0}^{N-1} [\beta_{\min,i}, \beta_{\max,i}], \tag{11}$$

and each subangular range spans $2\pi$, that is,

$$[\beta_{\min,i}, \beta_{\min,i}] = [0, 2\pi], \tag{12}$$

the original 3D weighted helical CB-FBP algorithm can be extended to reconstruct images from projection data corresponding to an overscan in the way specified below:

$$f(x, y, z) = \frac{1}{2N} \sum_{i=0}^{N-1} \int_{\beta_{\min,i}}^{\beta_{\max,i}} \frac{R}{\sqrt{R^2 + Z(x, y, z)^2}} w_{3d,i}(\alpha, \beta_i, t)\tilde{s}(\alpha, \beta_i, t)\, d\beta_i, \tag{13}$$

where the support of each 3D weighting function $w_{3d,i}(\alpha, \beta_i, t)$ is $[\beta_{\min,i}, \beta_{\max,i}]$, that is, view angle $\beta_i$ has to be determined within each subangular range, respectively.

Since a filtered backprojection reconstruction algorithm is linear from the perspective of system analysis, (13) can be rearranged as

$$f(x, y, z) = \frac{1}{2} \int_{\beta_{\min}}^{\beta_{\max}} \frac{R}{\sqrt{R^2 + Z(x, y, z)^2}} w_{3d}(\alpha, \beta, t)\tilde{s}(\alpha, \beta, t)\, d\beta, \tag{14}$$

with

$$w_{3d}(\alpha, \beta, t) = \frac{1}{N} \sum_{i=0}^{N-1} w_{3d,i}(\alpha, \beta_i, t), \tag{15}$$

$$w_{3d,i}(\alpha, \beta, \gamma) = \frac{w_{2d}(\beta_i, t) \tan^{kh}(\alpha_c)}{w_{2d}(\beta_i, t) \tan^{kh}(\alpha_c) + w_{2d}(\beta_{i,c}, t_c) \tan^{kh}(\alpha)}. \tag{16}$$

This means that the reconstructed image under a helical overscan is just a linear summation of the images reconstructed from data acquired over a series of full scans corresponding to each subangular range $[\beta_{\min,i}, \beta_{\max,i}]$ $(\beta_{\max,i} - \beta_{\min,i} = 2\pi)$. The reconstruction accuracy corresponding to a full scan $[\beta_{\min,i}, \beta_{\max,i}]$ $(\beta_{\max,i} - \beta_{\min,i} = 2\pi)$ has been evaluated and verified in [6]. Consequently, the reconstruction accuracy of the extended 3D weighted helical CB-FBP algorithm specified by (13) or (14) is warranted as long as the overscan angular range $[\beta_{\min,o}, \beta_{\max,o}]$ $(\beta_{\max,o} - \beta_{\min,o} = 2\pi + \Delta\beta)$ is decomposed into subangular ranges appropriately. Note that $N$ is a parameter that can be optimized under various low helical pitches to achieve a balance between the most achievable image quality and computational efficiency, and $w_{2d}(\beta_i, t)$ can be in the form [12]

$$w_{2d}(\beta_i, t) = \begin{cases} 0.0, & \beta \le -\pi, \\ 0.25 \cdot \dfrac{(\beta + \pi)}{\beta_t}, & -\pi < \beta \le -\pi + 2\beta_t, \\ 0.5, & -\pi + 2\beta_t < \beta \le -2\beta_t, \\ 0.5 + \dfrac{0.25(\beta + 2\beta_t)}{\beta_t}, & -2\beta_t < \beta \le 0, \\ 1.0 - \dfrac{0.25\beta}{\beta_t}, & 0 < \beta \le 2\beta_t, \\ 0.5, & 2\beta_t < \beta \le \pi - 2\beta_t, \\ 0.5 - 0.25 \cdot \dfrac{(\beta - \pi + 2\beta_t)}{\beta_t}, & \pi - 2\beta_t < \beta \le \pi, \\ 0.0, & \beta > \pi, \end{cases} \tag{17}$$

where $\beta_t$ is the parameter that can be adjusted to optimize the capability of suppressing artifacts and maintenance of noise characteristics or dose efficiency.

### 2.5. Evaluation

Just like the original algorithm, the extended 3D weighted helical CB-FBP algorithm is essentially approximate. Hence, the reconstruction accuracy of the extended algorithm has to be evaluated and verified experimentally. Two computer-simulated phantoms are utilized to evaluate the reconstruction accuracy. The first phantom is the helical body phantom (HBP) [14] to evaluate the reconstruction accuracy in transverse view, and the second is a modified Defrise phantom consisting of 5 cylindrical discs [15], rather than ellipsoidal discs in the original Defrise phantom, to evaluate the reconstruction accuracy in coronal view. The dimension of each disc is $200 \times 200 \times 10\,\text{mm}^3$, centered at the $z$-axis, and their linear attenuation coefficient is equivalent to a CT number of 600 HU in reconstructed tomographic images. The central disc is located at $z = 0.0$ mm, and other 4 discs are 20 mm apart along the $z$-direction. In the computer simulation, the distance from source to the iso-center of the volumetric CT is 541.0 mm, the X-ray detector consists of 64 detector rows and each detector row is made up of 888 detector cells with a dimension of 0.625 mm along $z$-axis and 0.5836 mm in $xy$-plane at the iso-center, respectively. The scanning techniques for the computer phantom simulation are 120 kVp, 300 mA, and 1.0 sec/rot, respectively. To quantitatively investigate noise characteristics or dose efficiency, a 20 cm water phantom scanned by a volumetric CT scanner (LightSpeed VCT, GE Healthcare, Waukesha, WI, USA) is employed. The matrix dimension of a reconstructed transverse image is $512 \times 512$, no matter the projection data is

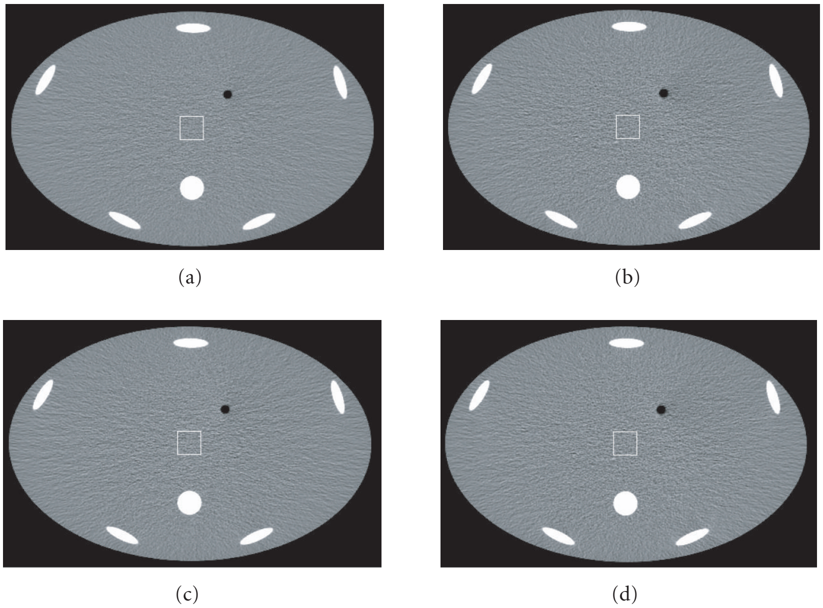

Figure 3: Tomographic images of the helical body phantom reconstructed by (a) the original algorithm using full-scan data at helical pitch 33/64 : 1; (b) the extended algorithm using overscan data at helical pitch 33/64 : 1; (c) the original algorithm using full-scan data at helical pitch 63/64 : 1; and (d) the extended algorithm using overscan data at helical pitch 63/64 : 1 (w/l = 200/0 HU).

simulated or scanned by the volumetric CT system, and 984 projections are simulated or scanned in one turn of the helical scanning (360°).

### 3. RESULTS

In general, the extended 3D weighted helical CB-FBP algorithm is applicable at any helical pitch lower than 1 : 1. Due to space limitation, only the results corresponding to helical pitch 33/64 : 1 and 63/64 : 1 are presented here. At helical pitch 33/64 : 1, the parameters of the weighting function are selected as $\beta_t = 0.15\pi$, $\beta_{\max} - \beta_{\min} = 2.5\pi$, and $N = 3$, while the parameters of the weighting function are selected as $\beta_t = 0.225\pi$, $\beta_{\max} - \beta_{\min} = 2.5\pi$ and $N = 3$, at helical pitch 63/64 : 1.

### 3.1. Evaluation of reconstruction accuracy

#### 3.1.1. Evaluation by the HBP phantom

At helical pitch 33/64 : 1, the transverse image of the computer-simulated HBP phantom reconstructed by the original algorithm using full-scan projection data is presented in Figure 3(a), while the one at the same location but reconstructed by the extended algorithm using overscan projection data is in Figure 3(b). The reconstruction field of view (FOV) is 450 mm, and no helical artifact is observed in both images, showing that the reconstruction accuracy of the extended algorithm is as good as that of the original algorithm. More specifically, it has to be revealed that $kh = 0.25$ for the original algorithm using full-scan projection data, while $kh = 0.125$ for the extended algorithm using overscan projection data to reconstruct the image.

At helical pitch 63/64 : 1, the transverse image of the HBP phantom reconstructed by the original algorithm using full-scan projection data is presented in Figure 3(c), while that at the same location but reconstructed by the extended algorithm using overscan projection data is in Figure 3(d). Again, no helical artifact is observed in both images, showing that the reconstruction accuracy of the extended algorithm is as good as that of the original algorithm at such a moderate helical pitch. Moreover, it has to be indicated that $kh = 0.5$ for both the extended and original algorithms using full-scan and overscan projection data, respectively.

#### 3.1.2. Evaluation by the Defrise phantom

Multiple planar reformatted images in the coronal view of the Defrise phantom reconstructed by the original algorithm at helical pitch 33/64 : 1 using full-scan projection data is shown in Figure 4(a), while that by the extended algorithm using overscan projection data in Figure 4(b). Meanwhile, the coronal view of the Defrise phantom reconstructed by the original algorithm at helical pitch 63/64 : 1 using full-scan projection data is shown in Figure 4(c), while that by the extended algorithm using overscan projection data is in Figure 4(d). Just like the original reconstruction algorithm, the extended algorithm can reconstruct the Defrise phantom very well, showing that its reconstruction accuracy is comparable to the original 3D weighted helical CB-FBP algorithm.

### 3.2. Evaluation of noise characteristics or dose efficiency

A total of 5 ROIs are chosen within the 250 mm FOV of the 20 cm cylindrical water phantom to gauge the noise

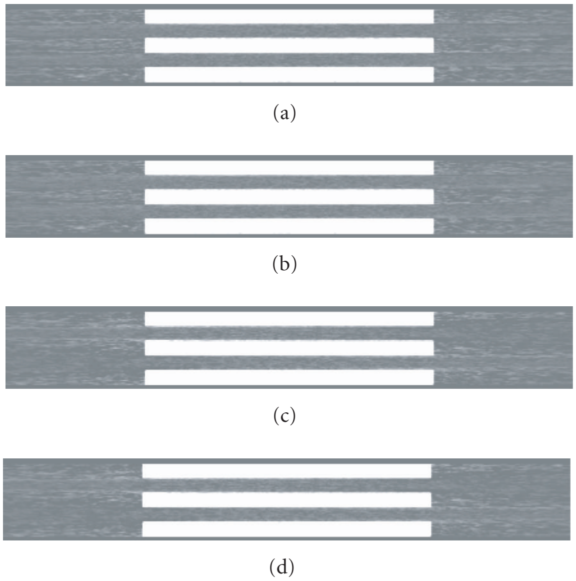

Figure 4: Multiple planar reformatted (coronal) images of the Defrise phantom reconstructed by (a) the original algorithm using full-scan data at helical pitch 33/64 : 1; (b) the extended algorithm using overscan data at helical pitch 33/64 : 1; (c) the original algorithm using full-scan data at helical pitch 63/64 : 1; and (d) the extended algorithm using overscan data at helical pitch 63/64 : 1 (w/l = 600/0 HU).

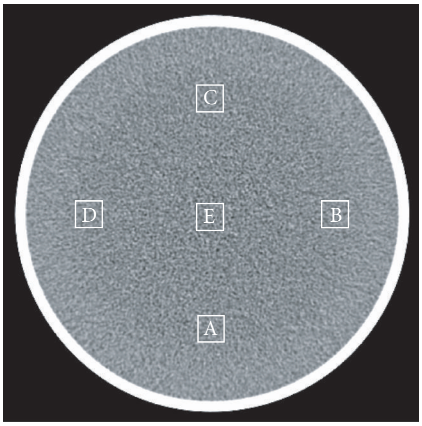

Figure 5: The schematic diagram showing the ROIs in the tomographic image of the 20 cm water phantom to gauge noise characteristics: ROI A, B, C, D, and E are square consisting of 30×30 pixels; the center of ROI A, B, C, and D is 67.5 mm from the iso, while the center of ROI E is at the iso.

characteristics. As shown in Figure 5, the ROI labeled by E is at the iso-center, while those ROIs labeled by A, B, C, and D, respectively, are located at 6, 3, 12, and 9 o'clock orientation at 62.5 mm from the iso-center. Each ROI contains 30 × 30 pixels, and the noise is measured as the standard deviation of Hounsfield Unit variation within the ROI.

At helical pitch 33/64 : 1, the measured noise at those ROIs corresponding to the original algorithm are itemized in the 1st column of Table 1, and those corresponding to the extended algorithm are in the 2nd column. Apparently, the noise level at each ROI in the image reconstructed by the extended algorithm is significantly smaller than that in the image reconstructed by the original algorithm. Moreover, the noise uniformity at those ROIs in the image reconstructed by the extended algorithm is significantly better than that in the images reconstructed by the original algorithm. However, as shown in the right columns of Table 1, there is virtually no improvement in noise level and uniformity at helical pitch 63/64 : 1 in the images reconstructed by the extended algorithm over the original algorithm. This is consistent with our observation in Section 2.2, in which it has been shown that there is almost no extra projection data in comparison to a full scan can be utilized to reconstruct an image at helical pitch 63/64 : 1.

Table 1: Noise measurement of the ROIs specified in Figure 5 at helical pitches 33/64 : 1 and 63/64 : 1, respectively.

| ROI | Pitch = 33/64 : 1 | | Pitch = 63/64 : 1 | |
|---|---|---|---|---|
| | Full scan ($kh = 0.125$) | Overscan ($kh = 0.125$) | Full scan ($kh = 0.5$) | Overscan ($kh = 0.5$) |
| A | 7.98 | 7.42 | 8.40 | 8.23 |
| B | 7.89 | 7.31 | 8.49 | 8.27 |
| C | 7.51 | 7.28 | 8.09 | 7.95 |
| D | 7.14 | 6.74 | 7.61 | 7.49 |
| E | 8.89 | 8.58 | 9.49 | 9.34 |

### 4. DISCUSSIONS AND CONCLUSIONS

It is well recognized that the 3D backprojection is the most computationally expensive process in image reconstruction using CB-FBP algorithms. It is important to point out that, the rearrangement of (13) into (14) not only simplifies algorithm expression but also improves image generation efficiency. In the implementation of (13), a total of $N$ images corresponding to projection data acquired along each subangular range and weighted by $w_{3d,i}(\alpha,\beta_i,t)$ have to be reconstructed using the original algorithm. However, in the implementation of (14), only one 3D backprojection is demanded to reconstruct image from projection data acquired along $[\beta_{\min,i},\beta_{\max i}]$ ($\beta_{\max,i}-\beta_{\min,i}=2\pi$) and weighted by $w_{3d}(\alpha,\beta,t)$. Apparently, such an $N:1$ ratio in computational complexity assures that the implementation of the extended 3D weighted helical CB-FBP algorithm by (14) is a better choice than that by (13) in practice.

It is worthwhile indicating that (11) and (12) do not demand each subangular range $[\beta_{\min,i},\beta_{\max,i}]$ ($\beta_{\max,i}-\beta_{\min,i}=2\pi$) evenly shifting in the decomposition to cover the whole angular range $[0,2\pi+\Delta\beta]$, while an even shifting can result in data manipulation efficiency. Moreover, it is not mandatory for the 3D weighting function $w_{3d}(\alpha,\beta,t)$ corresponding to $[\beta_{\min,o},\beta_{\max,o}]$ ($\beta_{\max,o}-\beta_{\min,o}=2\pi+\Delta\beta$) to be the average of each 3D weighting function $w_{3d-i}(\alpha,\beta_i,t)$ corresponding to $[\beta_{\min,i},\beta_{\max,i}]$ ($\beta_{\max,i}-\beta_{\min,i}=2\pi$) as specified by (15). Indeed, other weighted summation strategies can be exercised in practice.

As indicated in [6], the original 3D weighted helical CB-FBP algorithm can be implemented in the native CB geometry, although its derivation in the cone-parallel geometry was given as an example in [6]. Hence, the extended 3D weighted helical CB-FBP algorithm can also be implemented in the native CB geometry as its counterpart. Moreover, it has to be pointed out that the determination of the parameters $\Delta\beta$ and $kh$ is not optimized in the experimental evaluation shown above. All the parameters chosen are just to show how the extended 3D weighted helical CB-FBP algorithm works in overscan corresponding to helical pitches lower than 1 : 1.

It has to be emphasized that both the original 3D weighting scheme proposed in [6] and the extended 3D weighting scheme presented in this manuscript are ray-wise weightings. This means that, by making use of the cone angle corresponding to both direct and conjugate rays, the 3D weighting is actually a ray-wise optimization process to obtain balanced image quality and most achievable dose efficiency over helical pitches. By making use of the cone angle information corresponding to conjugate rays, our ray-wise 3D weighting scheme distinguishes itself from other 3D weighting schemes existing in the literature [7–9]. The readers that are interested in the difference between our ray-wise 3D weighting scheme and that proposed in [7] are referred to [6], where a brief discussion on the difference is given. Detector-row-dependent weighting schemes have been proposed in [8, 9], respectively. By using predefined weighting distribution, the ray intercepting inner detector rows are given favorable weights and those intercepting outer detector rows are given unfavorable weights, in which the cone angle information corresponding to conjugate ray is not utilized. The weight in our method is ray-wise calculated and such a ray-wise optimization process can make use of redundant projection data as much as possible over helical pitches, particularly at high helical pitches. However, it seems very hard, if not impossible, to make use of projection data as much as possible by using a predefined weighting distribution in the detector as done in [8, 9]. Interested readers are referred to references [8, 9] for more detail.

Moreover, it should be indicated that, the extended 3D weighted helical CB-FBP algorithm proposed in this manuscript is quite different from the n-PI CB reconstruction methods existing in the literature [25–27]. The concepts of Tam-window, PI-line, N-PI window, and N-PI line play critical roles in the derivation of both exact and approximate n-PI CB reconstruction algorithms. However, as clearly shown above, none of these concepts has been utilized in deriving the extended 3D weighted helical CB-FBP algorithm.

In summary, recognizing the clinical importance of reconstructing tomographic images from overscan projection data, the original 3D weighted helical CB-FBP algorithm has been extended in this manuscript to reconstruct tomographic images at helical pitches lower than 1 : 1. As shown in previous sections, such an extension can make use of projection data as efficient as possible, resulting in significantly improved noise characteristics or dose efficiency. In principle, the extended 3D weighted helical CB-FBP algorithm is approximate, but its reconstruction accuracy has been evaluated and verified at a relatively moderate cone angle ($4^\circ$) corresponding to a detector with the dimension of $64 \times 0.625$ mm at the source to iso-center distance of 541.0 mm. In fact, the extended algorithm is expected to be applicable in volumetric CT scanners with the detector $z$-dimension corresponding to larger cone angles, and more investigation is being carried out to assure the maximum cone angle up to which the proposed algorithm can still provide acceptable reconstruction accuracy for diagnostic CT imaging applications.

### ACKNOWLEDGMENT

The authors would like to express their appreciation to the anonymous reviewers for their constructive comments and suggestions.

### REFERENCES

[1] A. Katsevich, "Analysis of an exact inversion algorithm for spiral cone-beam CT," *Physics in Medicine and Biology*, vol. 47, no. 15, pp. 2583–2597, 2002.

[2] A. Katsevich, "Theoretically exact filtered backprojection-type inversion algorithm for spiral CT," *SIAM Journal on Applied Mathematics*, vol. 62, no. 6, pp. 2012–2026, 2002.

[3] Y. Zou and X. Pan, "Exact image reconstruction on PI-lines from minimum data in helical cone-beam CT," *Physics in Medicine and Biology*, vol. 49, no. 6, pp. 941–959, 2004.

[4] J. D. Pack and F. Noo, "Cone-beam reconstruction using 1D filtering along the projection of M-lines," *Inverse Problems*, vol. 21, no. 3, pp. 1105–1120, 2005.

[5] G.-H. Chen, "An alternative derivation of Katsevich's cone-beam reconstruction formula," *Medical Physics*, vol. 30, no. 12, pp. 3217–3226, 2003.

[6] X. Tang, J. Hsieh, R. A. Nilsen, S. Dutta, D. Samsonov, and A. Hagiwara, "A three-dimensional-weighted cone beam filtered backprojection (CB-FBP) algorithm for image reconstruction in volumetric CT - Helical scanning," *Physics in Medicine and Biology*, vol. 51, no. 4, pp. 855–874, 2006.

[7] K. Taguchi, B.-S. S. Chiang, and M. D. Silver, "A new weighting scheme for cone-beam helical CT to reduce the image noise," *Physics in Medicine and Biology*, vol. 49, no. 11, pp. 2351–2364, 2004.

[8] K. Stierstorfer, A. Rauscher, J. Boese, H. Bruder, S. Schaller, and T. Flohr, "Weighted FBP—a simple approximated 3D FBP algorithm for multislice spiral CT with good dose usage for arbitrary pitch," *Physics in Medicine and Biology*, vol. 49, no. 11, pp. 2209–2218, 2004.

[9] D. Heuscher, K. Brown, and F. Noo, "Redundant data and exact helical cone-beam reconstruction," *Physics in Medicine and Biology*, vol. 49, no. 11, pp. 2219–2238, 2004.

[10] I. A. Feldkamp, L. C. Davis, and J. W. Kress, "Practical cone-beam algorithm," *Journal of the Optical Society of America A: Optics and Image Science, and Vision*, vol. 1, no. 6, pp. 612–619, 1984.

[11] G. Wang, T.-H. Lin, P.-C. Cheng, and D. M. Shinozaki, "General cone-beam reconstruction algorithm," *IEEE Transactions on Medical Imaging*, vol. 12, no. 3, pp. 486–496, 1993.

[12] C. R. Crawford and K. F. King, "Computed tomography scanning with simultaneous patient translation," *Medical Physics*, vol. 17, no. 6, pp. 967–982, 1990.

[13] M. D. Silver, "A method for including redundant data in computed tomography," *Medical Physics*, vol. 27, no. 4, pp. 773–774, 2000.

[14] X. Tang, "Matched view weighting in tilted-plane-based reconstruction algorithms to suppress helical artifacts and optimize noise characteristics," *Medical Physics*, vol. 30, no. 11, pp. 2912–2918, 2003.

[15] X. Tang and J. Hsieh, "A filtered backprojection algorithm for cone beam reconstruction using rotational filtering under helical source trajectory," *Medical Physics*, vol. 31, no. 11, pp. 2949–2960, 2004.

[16] M. Defrise and R. Clack, "Cone-beam reconstruction algorithm using shift-variant filtering and cone-beam backprojection," *IEEE Transactions on Medical Imaging*, vol. 13, no. 1, pp. 186–195, 1994.

[17] P. E. Danielsson, P.-E. P. Edholm, J. Eriksson, and M. Magnusson-Seger, "Towards exact 3D-reconstruction for helical cone-beam scanning of long objects: a new arrangement and a new completeness condition," in *International Meeting on Fully Three-Dimensional Image Reconstruction in Radiology and Nuclear Medicine*, pp. 141–144, Pittsburgh, Pa, USA, June 1997.

[18] S. Schaller, T. Flohr, and P. Steffen, "New efficient Fourier-reconstruction method for approximate image reconstruction in spiral cone-beam CT at small cone angles," in *Medical Imaging 1997: Physics of Medical Imaging*, vol. 3032 of *Proceedings of SPIE*, pp. 213–224, Newport Beach, Calif, USA, February 1997.

[19] H. Tuy, "3D image reconstruction for helical partial cone beam scanners using wedge beam transform," Patent, US 6,104,775, 2000.

[20] X. Yan and R. M. Leahy, "Cone beam tomography with circular, elliptical and spiral orbits," *Physics in Medicine and Biology*, vol. 37, no. 3, pp. 493–506, 1992.

[21] D. L. Parker, "Optimal short scan convolution reconstruction for fanbeam CT," *Medical Physics*, vol. 9, no. 2, pp. 254–257, 1982.

[22] J. Hsieh, "A general approach to the reconstruction of x-ray helical computed tomography," *Medical Physics*, vol. 23, no. 2, pp. 221–229, 1996.

[23] K. Taguchi and H. Aradate, "Algorithm for image reconstruction in multi-slice helical CT," *Medical Physics*, vol. 25, no. 4, pp. 550–561, 1998.

[24] H. Hu, "Multi-slice helical CT: scan and reconstruction," *Medical Physics*, vol. 26, no. 1, pp. 5–18, 1999.

[25] R. Proska, Th. Koehler, M. Grass, and J. Timmer, "The n-PI-method for helical cone-beam CT," *IEEE Transactions on Medical Imaging*, vol. 19, no. 9, pp. 848–863, 2000.

[26] A. Katsevich, "On two versions of a $3\pi$ algorithm for spiral CT," *Physics in Medicine and Biology*, vol. 49, no. 11, pp. 2129–2143, 2004.

[27] H. Yu, Y. Ye, S. Zhao, and G. Wang, "A backprojection-filtration algorithm for nonstandard spiral cone-beam CT with an n-PI-window," *Physics in Medicine and Biology*, vol. 50, no. 9, pp. 2099–2111, 2005.

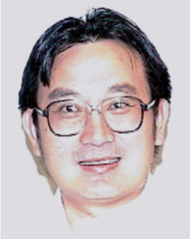

**Xiangyang Tang** is currently with the Applied Science Laboratory of GE Healthcare Technologies as a Senior Scientist. With a focus on the development of image reconstruction algorithms for cone beam (CB) volumetric computed tomography (VCT) in diagnostic imaging, he has been working in the field of CB-VCT for almost 10 years, covering system analysis and design, development of algorithms for system calibration, artifacts suppression, digital image/video processing, and CT cardiac imaging applications. In the recent 5 years, as an active Researcher in the field of medical imaging, he published more than 50 papers or book chapters in scientific journals and conference proceedings, and filed more than 10 US patent applications. Meanwhile, he also served as Guest Editor or Reviewer for a number of prestigious scientific journals in the field of medical imaging.

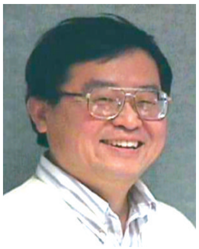

**Jiang Hsieh** is a Chief Scientist in the Applied Science Laboratory of GE Healthcare Technologies and an adjunct Professor in the Medical Physics Department of the University of Wisconsin, Madison. He has more than 20 years of experience in medical imaging. He holds over 100 US patents, has coauthored more than 100 articles, book chapters, and textbook. He taught AAPM summer school, refresher courses at RSNA, short courses at IEEE Medical Imaging Conference, AAPM annual meeting, and SPIE Medical Imaging Conference. His research interests include tomographic reconstruction, CT image artifact reduction and correction, signal processing, image processing, and advanced CT applications.

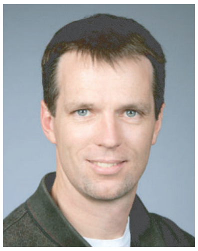

**Roy A. Nilsen** is currently a Principal Engineer of the Functional Imaging and Computed Tomography Image Reconstruction Group of GE Healthcare Technologies. He has been developing SW and HW for General Electric medical imaging devices since June 1988. Most of that time has been spent developing image reconstruction modules for CT systems. He has a B.S. degree in both electrical engineering and computer science from the University of Wisconsin.

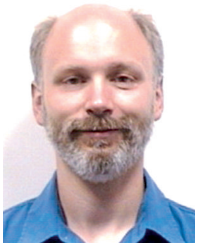

**Scott M. McOlash** is a software Engineer in the Functional Imaging and Computed Tomography Image Reconstruction Group of GE Healthcare Technologies, where he currently develops and integrates reconstruction algorithms. His career includes 20 years of hardware and software development in applied research, CAD/CAM integration, industrial control networking, and diagnostic imaging. He holds an M.S. degree and a B.S. degree in electrical engineering from Marquette University in Milwaukee, WI.

